# Vitamin C as a Modulator of the Response to Cancer Therapy

**DOI:** 10.3390/molecules24030453

**Published:** 2019-01-28

**Authors:** Wiktoria Blaszczak, Wojciech Barczak, Julia Masternak, Przemysław Kopczyński, Anatoly Zhitkovich, Błażej Rubiś

**Affiliations:** 1 Radiobiology Lab, The Greater Poland Cancer Centre, Garbary, 61-866 Poznan, Poland; w.z.blaszczak@gmail.com (W.B.); wbarczak@ump.edu.pl (W.B.); 2 Department of Head and Neck Surgery, Poznan University of Medical Sciences, The Greater Poland Cancer Centre, Garbary, 61-866 Poznan, Poland; 3 Department of Clinical Chemistry and Molecular Diagnostics, Poznan University of Medical Sciences, 60-355 Poznan, Poland; juliamasternak@wp.pl; 4 Centre for Orthodontic Mini-implants at the Department and Clinic of Maxillofacial Orthopedics and Orthodontics, Poznan University of Medical Sciences, 60-812 Poznan, Poland; pkopczynski@orto1.net; 5 Department of Pathology and Laboratory Medicine, Brown University, Providence, RI 02912, USA; anatoly_zhitkovich@brown.edu

**Keywords:** vitamin C, ascorbate, cancer, cancer therapy, chemotherapy, oxidative stress, ROS, hypoxia

## Abstract

Ascorbic acid (vitamin C) has been gaining attention as a potential treatment for human malignancies. Various experimental studies have shown the ability of pharmacological doses of vitamin C alone or in combinations with clinically used drugs to exert beneficial effects in various models of human cancers. Cytotoxicity of high doses of vitamin C in cancer cells appears to be related to excessive reactive oxygen species generation and the resulting suppression of the energy production via glycolysis. A hallmark of cancer cells is a strongly upregulated aerobic glycolysis, which elevates its relative importance as a source of ATP (Adenosine 5′-triphosphate). Aerobic glycolysis is maintained by a highly increased uptake of glucose, which is made possible by the upregulated expression of its transporters, such as GLUT-1, GLUT-3, and GLUT-4. These proteins can also transport the oxidized form of vitamin C, dehydroascorbate, permitting its preferential uptake by cancer cells with the subsequent depletion of critical cellular reducers as a result of ascorbate formation. Ascorbate also has a potential to affect other aspects of cancer cell metabolism due to its ability to promote reduction of iron(III) to iron(II) in numerous cellular metalloenzymes. Among iron-dependent dioxygenases, important targets for stimulation by vitamin C in cancer include prolyl hydroxylases targeting the hypoxia-inducible factors HIF-1/HIF-2 and histone and DNA demethylases. Altered metabolism of cancer cells by vitamin C can be beneficial by itself and promote activity of specific drugs.

## 1. Introduction

The potential use of intravenous ascorbic acid (AA) as a complementary agent in cancer treatment has been studied since the 1970s [1,2,3]. Vitamin C has been explored as a component of combination therapy, either because of its synergy with primary treatment and chemosensitization activity [4,5] or, surprisingly, cancer cell death suppression agent [6,7]. Although at physiological levels vitamin C acts as an antioxidant, its therapeutic effectiveness at pharmacological doses in some cases appears to be linked to pro-oxidant effects ultimately promoting cancer cell death [8]. Furthermore, recent studies identified AA as a potent epigenetic regulator, thus making it a potential complementary agent in the application of the emerging therapy targeting cancer epigenome [9,10,11,12]. Use of AA in cancer management is supported by vitamin C deficiency observed in many cancer patients [13,14,15,16], including those suffering from the most aggressive forms of the disease where deficiency is even more severe [17].

Cultured cells are the main biological models for most biochemical and other molecular studies aimed at understanding of normal and malignancy-associated metabolism, growth, and regulatory networks. One well-recognized disadvantage of the typical cell culture is its limited ability to recapitulate a multicellular context of tissues. Another concern is the supply of the physiologically relevant concentrations of nonessential nutrients in growth media, which can significantly alter physiology of cells. One of such nutrients is vitamin C. Most standard cell culture formulations do not include vitamin C and its only source for cells in culture is the addition of serum (fetal bovine or other). Since serum is typically added at 10% and AA undergoes irreversible oxidation during storage, the concentrations of vitamin C even in freshly fed cells are in low micromolar range versus low millimolar levels in vivo [18,19]. Vitamin C can be rapidly delivered into cells by the addition of its oxidized form, dehydroascorbic acid (DHA), to the low-glucose medium with or without serum followed by a change to a regular medium. A more gradual cellular accumulation of vitamin C occurs when cell culture medium is supplemented with AA or its more stable derivative, ascorbate-phosphate. Restoration of physiological levels of ascorbate can dramatically change how cells respond to redox-active DNA-damaging agents [20].

Recognition of vitamin C deficiency in cell culture varies between the fields of study and currently it is rarely addressed in in vitro studies relevant to cancer biology and therapy. Similar to guinea pigs and apes, humans have a mutationally inactivated l-gluconolactone oxidase gene (*GULO*), which codes for the enzyme that catalyzes the final step in the biosynthesis of vitamin C [21]. Thus, AA must be provided to these species with food. Importantly, rodent models that possess the ability to synthesize vitamin C do not fully recapitulate the effects of vitamin C supplementation in humans. [22]. *Gulo*−/− knockout mouse strain represents a more faithful biological model for studies of human vitamin C metabolism and these animals develop normally and healthy with dietary supplementation of AA [23]. Additional models of vitamin C deficiency include guinea pig and the osteogenic disorder Shionogi strain of rats [22]. Vitamin C deficiency in animals causes multiple pathologies, including partial loss of neutrophil function, increased oxidative stress during development [24], neuroprotection failure and neurotransmission disorders, and, eventually, death [25]. Use of animal models with the loss of *Gulo* activity offers the opportunities to experimentally test the contribution of vitamin C to growth, metabolism, and responses of human cancers to therapeutics.

## 2. Vitamin C

Vitamin C is present in the bloodstream at approximately 50–100 μM concentration in plasma of healthy subjects [26]. Human blood cells also contain AA, which is delivered through the activity of different transporters for reduced or oxidized forms of vitamin C: sodium-dependent vitamin C transporters (SVCT 1 and 2) for AA transport or GLUT1, 3, and 4 for DHA entry [27,28]. Dietary consumption of vitamin C results in lower plasma levels of ascorbate than intravenous injection, but the excess of AA in the blood is transient due to its efficient excretion in the urine. In vivo concentrations of vitamin C showed a significant variation among tissues. In *Gulo* knockout mice, brain and heart were found to accumulate higher levels of AA than other organs [29]. Once inside the cells, DHA is rapidly reduced to AA that exerts various effects on cell metabolism. At physiological concentrations, AA is known for its antioxidant properties (by scavenging free radicals) and its importance in collagen synthesis as a cofactor in the enzymatic hydroxylation of lysine and proline residues. Reduced vitamin C is estimated to serve as a cofactor for approximately 150 human enzymes [30,31,32], indicating a much broader impact of AA on cell and tissue physiology. Ascorbate functions as a metal-reducing cofactor for many enzymes, including copper-containing monooxygenases and Fe(II+)/2-oxoglutarate (2OG)-dependent dioxygenases. For example, ascorbate is involved in the regulation of hypoxia-inducible factors’ (HIF-1α and HIF-2α) stability, via their prolyl-hydroxylation, and in the epigenetic control of gene expression, via demethylation of histone lysines and CpG sites in DNA [33].”

Consistent with its role in genome transcription, vitamin C was found to upregulate the expression of a series of genes that contribute to energy metabolism, immune responses, and cytoskeleton formation [34].

### 2.1. Vitamin C in Cancer Treatment

High-dose vitamin C has been studied as a potential cancer treatment since the 1970s [1]. Results from more recent clinical trials showed that intravenous vitamin C was safe in cancer patients, producing minimal side effects [35]. However, while generally considered as a dietary supplement, neither the U.S. Food and Drug Administration nor European Medicines Agency has approved the use of intravenous high-dose vitamin C as a treatment for cancer. Vitamin C has been shown to diminish the effects of chemotherapy due to its antioxidant properties when applied in low/physiological concentrations [6,7]. Other data indicate that combining high-dose vitamin C with anticancer therapies inhibits tumor growth in models of pancreatic [36,37], liver [38], prostate [39], ovarian cancer [40], sarcoma [41] and malignant mesothelioma [41]. Furthermore, several trials of high-dose intravenous vitamin C administration in cancer patients have led to increased quality of life, as well as improvements in physical, mental, and emotional functions, and less frequent adverse effects including fatigue, nausea, vomiting, pain, and appetite loss [33,42]. However, many questions regarding the potential interactions between AA and chemotherapy depending on the dosing regiments remain unaddressed. Clinical studies have shown that in pre-screened patients with advanced solid tumors intravenous administration of vitamin C was well tolerated even at doses up to 1.5 g/kg of body weight or 70–80 g/m^2^ [38]. It was also reported that breast cancer patients [39], as well as metastatic pancreatic cancer patients [40], experienced less severe chemotherapy-induced side effects after a complementary intravenous AA treatment.

Pires et al. found that simultaneous administration of ascorbate with oxaliplatin or irinotecan inhibited tumor growth in vivo, and the effect was significantly higher compared to that of these compounds alone [43]. AA was also reported as a potent chemosensitizer to gefitinib-based therapy in non-small cell lung cancer [44]. Another study of high AA dosage on ovarian cancer cells observed induction of DNA damage, depletion of cellular ATP, and activation of the corresponding stress signaling kinases, ATM (ataxia telangiectasia mutated) and AMPK (AMP-activated protein kinase). The resulting repression of mTOR led to death of cancer cells [45]. Importantly, authors also showed that the combination of parenteral AA with the conventional chemotherapeutic agents carboplatin and paclitaxel synergistically inhibited ovarian cancer in mouse models and reduced chemotherapy-associated toxicity in patients with ovarian cancer. In vitro studies with a different cell model also detected synergistic effects of AA cotreatment with carboplatin and paclitaxel [46].

Physiological levels of vitamin C efficiently detoxify reactive oxygen species (ROS) and reactive nitrogen species that are formed during normal metabolism but frequently overproduced under various forms of stress [41]. Consequently, vitamin C is protective against cell injury and death by pro-oxidant stressors [39]. It is likely that at least partially, reduction in toxicity of several chemotherapeutic agents on normal tissue upon co-administration with AA is related to suppression of a collateral oxidative damage in nontarget cells [47,48].

### 2.2. HIF and GLUT Links

Most solid tumors contain regions of hypoxia, which appear as a result of limited blood supply. To survive under stressful hypoxic conditions, tumor cells activate critical adaptation mechanisms. A clinical consequence of this metabolic remodeling is a heightened aggressiveness of the disease, which is manifested by resistance to therapy and decreased patient survival. A crucial mediator of the hypoxic response is the transcription factors HIF-1 and HIF-2. Together, these factors upregulate the expression of hundreds of genes that upregulate angiogenesis, glucose uptake, anaerobic metabolism, and cell motility [49]. Interestingly, intracellular ascorbate suppresses the transcriptional activity of HIF-1 and HIF-2. This repressive effect was attributed to stimulation of HIF-1 degradation by ascorbate under normoxic or mildly hypoxic conditions by supporting the activity of iron-dependent-dioxygenases that hydroxylate critical Pro and Asn residues in the HIF-1α subunit [24,50,51]. AA at physiological concentrations significantly suppressed HIF-1α levels and expression of HIF-1 transcriptional targets in cancer cell lines [52]. However, the intracellular ascorbate content in many aggressive cancers may be suboptimal for the effective HIF-1 control [52,53,54], which can potentially be remediated by administration of pharmacological doses of vitamin C. Upregulation of HIF-1 and the hypoxia-like transcription response by two frequently used hypoxia mimetics cobalt and nickel has been suppressed by the supplementation with AA [55,56].

Metabolic reprogramming of all transformed cells is associated with overexpression of glucose transporters such as GLUT-1, GLUT-3 or GLUT-4 (glucose transporters 1, 3, and 4, respectively). GLUT and glycolysis genes are also positively regulated by HIF-1 as a part of the cellular adaptation to the low-nutrient conditions and high growth conditions. Thus, glucose uptake and glycolytic metabolism are enhanced in cancer compared to normal cells [57]. Interestingly, transport of an oxidized form of ascorbate, DHA, is mediated by GLUT transporters [58,59,60,61]. In normal cells, GLUTs are unlikely to play a major role in the uptake of vitamin C due to competition from much higher concentrations of glucose. The overabundance of GLUT transporters in cancer cells diminishes the inhibitory effects of glucose, permitting a higher accumulation of cellular vitamin C through uptake of DHA [62]. Regardless of glucose levels, a high expression of GLUT transporters was associated with the more efficient transport of vitamin C into the cells. Thus, both SVCT1,2 and GLUT transporters may collaborate in increasing the intracellular vitamin C concentration in cancer cells. Down-regulation of SVCT levels through a negative feedback mechanism [63] and a frequent presence of the alternative, inactive transcript of the widely expressed SVCT2 [64] lessens the significance of this transporter system for vitamin C uptake by many types of cells in favor of GLUTs. Thus, the heightened ability of GLUT-overexpressing cancer cells offers the opportunity to selectively overload them with ascorbate. This concept was directly tested in the study of colon cancer cells overexpressing GLUT-1 due to the presence of activating mutations in KRAS (Ki-ras2 Kirsten rat sarcoma viral oncogene homolog) or BRAF (B-Raf proto-oncogene, serine/threonine kinase) oncogenes [65]. The authors have found that high doses of vitamin C selectively killed colon cancer cells harboring mutated BRAF or KRAS through hyperaccumulation of its oxidized form, DHA, via GLUT-1. High DHA uptake caused a severe oxidative stress as a result of reduction of DHA to AA and the associated depletion of glutathione. The rise in ROS caused inactivation of the essential glycolytic enzyme GAPDH (glyceraldehyde-3-phosphate dehydrogenase), resulting in the energy crisis and cell death. Overall, this seminal study established a mechanistic rationale for the therapeutic applications of high vitamin C doses for cancers with two frequently mutated oncogenes.

Tumor toxicity studies performed in a gastric cancer model showed that cells with high GLUT1 (glucose transporter) expression were more sensitive to AA treatment than cells with low GLUT1 expression [66]. This observation is consistent with a contribution of GLUTs to vitamin C transport into the cells. A higher sensitivity of GLUT1-overexpressing cancer cells may not entirely be due to a higher cellular accumulation of vitamin C, as a greater glycolysis dependence or a modulation of enzymatic activities associated with the GLUT1-high phenotype could play a significant role in AA sensitivity. At high concentrations, AA can act as a pro-oxidant which in part results from its ability to effectively reduce Fe^3+^ and Cu^2+^ leading to elevated hydroxyl radical production in the Fenton reaction between Fe^2+^/Cu^+^ and H_2_O_2_ [67]. The reactivity of DHA and its degradation products with cellular proteins (dehydroascorbylation and glycation) is also potentially damaging and affecting gene expression [68].

### 2.3. Redox-Active Drugs

In most cases, the use of AA was considered as a part of strategy to exploit cancer vulnerabilities. However, AA may also directly influence the efficacy of a particular therapy through direct interactions with a redox-active drug. One logical candidate for such interactions is bleomycin. Bleomycin is a glycopeptide antitumor antibiotic produced by the bacterium *S. verticillus*, which has been used for the treatment of several diseases, including Hodgkin’s lymphoma, squamous cell carcinomas, testicular cancer, as well as in animal models of pulmonary fibrosis [69]. Its anticancer activity results from the formation of DNA single- and double-strand breaks, leading to tumor cell death [70]. Bleomycin is used in combination with doxorubicin in Hodgkin’s lymphoma. Doxorubicin acts by intercalating DNA bases and by inhibiting topoisomerase II to prevent its dissociation from DNA and resealing of enzymatically induced breaks in both strands. The effects of both drugs on the DNA are complementary [71]. Mechanism of action of bleomycin is associated with chelation of iron and a pseudoenzyme activity, which in the presence of oxygen produces superoxide and hydroxyl radicals that in turn cleave DNA [70,72]. Bleomycin activity is limited by Fe^2+^ accessibility that is influenced by the redox system. In light of its metal-reducing and antioxidant properties, AA may significantly contribute to the biological effects of bleomycin. Lymphocytes isolated from the blood of individuals supplemented with vitamin C (1 g per day for 4 weeks) showed a decreased amount of chromosome damage after exposure to bleomycin during in vitro [73]. A later study showed that bleomycin combined with vitamin C or beta-carotene affected endogenous hepatic antioxidant enzymes in rats (glutathione peroxidase/GPx, glutathione reductase, and glucose-6-phosphate dehydrogenase). Thus, it was concluded that bleomycin cytotoxicity was mediated through the generation of reactive oxygen species. However, both vitamins displayed differential effects on the enzyme activity suggesting that they impacted different processes [74].

DNA degradation activity of Fe-containing bleomycin is controlled by the availability of reducing agents [75,76]. At the same time, because cellular reducing factors such as AA can scavenge free radicals and other oxidative species, it has been assumed that such reducing agents would protect cells by suppressing the oxidative processes initiated by bleomycin. Buettner et al. demonstrated that both phenomena depend upon whether bleomycin reaching DNA before (DNA damage effect) or after (free radicals scavenging) its interaction with reducing agents [77]. The authors demonstrated that in the presence of DNA, AA initiated bleomycin-induced strand breaks whereas in the absence of DNA, AA reacted with bleomycin to produce the ascorbyl radical and a redox-inactive bleomycin that was incapable of nicking DNA. The authors suggested that reducing agents, such as AA, might protect cells from bleomycin toxicity by rendering bleomycin redox-inactive prior to its DNA binding.

Unfortunately, as it is the case with most cancer drugs, bleomycin has side effects. The most severe complication of bleomycin-based treatment is pulmonary fibrosis and impaired lung function. However, those effects might be abrogated by use of deglyco-bleomycin, which is a modified bleomycin-derived molecule in which the sugar residue D-mannosyl-L-glucose disaccharide has been removed [78]. Schroeder et al. showed that bleomycin was more potent than deglyco-bleomycin in the supercoiled plasmid DNA relaxation assay as well as in DU145 prostate cancer cells [79]. Additionally, the uptake assessment experiments (dye-labeled conjugates) found that the disaccharide moiety was critical for the tumor cell-targeting properties of bleomycin. These in vitro studies raised a concern that even if deglyco-BLM mediates DNA cleavage with a similar efficiency as bleomycin, it could exert a lower antitumor activity due to its diminished cellular uptake. However, experiments performed in three cancer models in rodents, including a human Hodgkin’s lymphoma xenograft and a syngeneic melanoma model, demonstrated that intraperitoneal administration of deglyco-bleomycin was as effective as bleomycin in inducing tumor regression. Moreover, whereas the antitumor effect of bleomycin was accompanied by a loss of body weight and the development of pulmonary toxicity, deglyco-bleomycin did not affect body weight and did not produce lung injury [78]. Both drugs induced lung epithelial cell apoptosis after intratracheal administration, but deglyco-bleomycin lost the ability to induce caspase-1 activation and the production of ROS, transforming growth factor-β1, and other profibrotic and inflammatory cytokines in the lungs of mice and in vitro. Deglyco-bleomycin should be considered for clinical testing as a less toxic alternative to bleomycin in cancer therapy.

## 3. Conclusions

The addition of high doses of AA alone or in combination with standard cancer drugs significantly enhances suppression of tumor growth. The efficacy of AA is strongly dependent on the route of its administration. When ascorbate is administered orally, only moderate increase in its plasma concentration is achieved. In contrast, when ascorbate is administered intravenously, concentrations in the millimolar levels are easily achieved although for a short period only. Thus, the intravenous administration of ascorbate can yield therapeutic plasma levels, while oral treatments are not effective [80,81,82]. Vitamin C exerts beneficial effects in cancer treatment through more than one mechanism, some of which are linked to the metabolism of transformed cells whereas others may involve direct interactions with specific drugs.

## Abbreviations

AA	ascorbic acid
BLM	bleomycin
DHA	l-dehydroascorbic acid
GLUT1	glucose transporter
HIF	hypoxia-inducible factor
ROS	reactive oxygen species
